# Blood urea nitrogen to albumin ratio and 1-year mortality after hip fracture surgery in older adults: a retrospective cohort study

**DOI:** 10.3389/fmed.2026.1877434

**Published:** 2026-07-17

**Authors:** Jian Wang, Liuyang Shi, Zihan Zhang, Jiale Guo, Junjie Xu

**Affiliations:** Department of Orthopedics, The Fourth Affiliated Hospital of Anhui Medical University, Hefei, China

**Keywords:** albumin, blood urea nitrogen, hip fracture, mortality, prognosis, risk factors

## Abstract

**Background:**

Hip fracture (HF) is a fragility fracture associated with a high risk of death in older adults. The blood urea nitrogen to albumin ratio (BAR) may reflect renal function and perfusion status, metabolic stress, and nutritional reserve, but its prognostic value in older patients with hip fracture remains unclear. This study aimed to evaluate the association between BAR and the risk of 1-year all-cause mortality after HF.

**Methods:**

We retrospectively included patients aged 60 years or older who underwent surgery for HF between May 2016 and December 2021. Several inflammation and nutrition related composite indicators were calculated using laboratory tests obtained early after admission. Logistic regression analysis was used to evaluate the associations between these indicators and 1-year mortality after HF. Restricted cubic spline (RCS) analysis, area under the receiver operating characteristic curve (AUC), sensitivity analysis, and subgroup analysis were further performed to assess the dose response relationship, predictive performance, and robustness of BAR.

**Results:**

A total of 521 patients were included, of whom 77 died within 1 year after hip fracture, corresponding to a mortality rate of 14.8%. In univariate analysis, SIRI, AISI, HALP, PNI, BAR, and NPAR were associated with mortality risk. After further adjustment for demographic characteristics, fracture related factors, surgical duration, and candidate confounders, only BAR remained consistently associated with mortality. In model 3, higher BAR was associated with an increased risk of 1-year all-cause mortality after hip fracture (OR = 1.13, 95% CI: 1.05–1.22, *P* < 0.01). RCS analysis did not show a significant nonlinear association. The AUC of BAR was 0.67, which was higher than that of BUN and ALB. No significant interaction was observed in subgroup analyses.

**Conclusion:**

Higher BAR early after admission was associated with an increased risk of 1-year mortality in older patients with hip fracture. BAR may serve as a simple and low-cost auxiliary indicator for risk stratification, although its predictive performance was moderate and requires further validation in multicenter prospective studies.

## 1 Introduction

Hip fracture (HF) is one of the most devastating fragility fractures in older adults, with clinical consequences that often extend far beyond the skeletal injury itself. As early as the late twentieth century, Cooper et al predicted that the number of HF would continue to rise with global population aging (1). Recent evidence from the Global Burden of Disease Study further showed that there were approximately 14.2 million patients with HF worldwide in 2019, with an age standardized incidence of about 182 per 100,000 population (2). At the individual level, the lifetime risk of HF approaches 20% in women and 10% in men (3). Beyond its substantial epidemiological burden, HF also leads to considerable health care expenditures and long-term care needs. A matched cohort study from Canada estimated that the incremental health system costs during the first year after HF were approximately 36,929 Canadian dollars for women and 39,479 Canadian dollars for men (4). HF has therefore become a major clinical and health economic challenge in aging societies (5).

Mortality is one of the most important long-term outcomes after HF. In a meta-analysis including older women and men, Haentjens et al found that the excess risk of death was greatest during the first 3 months after HF, with relative risks of approximately 5.75 in women and 7.95 in men compared with age matched individuals without HF; the excess mortality risk persisted for many years (6). In clinical cohorts, the 1-year mortality rate after HF may still reach or exceed 20% (7). Although early surgery, perioperative optimization, and orthogeriatric collaborative care may improve patient outcomes (8, 9), substantial heterogeneity in prognosis remains. Existing mortality prediction models differ considerably in variable selection, external validation, and clinical feasibility, which limits their use for rapid risk stratification early after admission (10).

Adverse outcomes in older patients with HF are usually driven by multiple factors, including advanced age, comorbidities, malnutrition, sarcopenia, frailty, anemia, renal dysfunction, and systemic inflammation (11–13). In recent years, inflammation and nutrition related composite indicators derived from routine blood tests have been investigated for prognostic assessment in patients with HF, including the neutrophil to lymphocyte ratio, systemic inflammation response index, and prognostic nutritional index (14–17). However, most available studies have focused on inflammatory cell counts or nutritional scores, whereas relatively less attention has been paid to simple indicators that may simultaneously reflect metabolic stress, renal function or perfusion status, and nutritional reserve.

The blood urea nitrogen to albumin ratio (BAR) is calculated from two routinely measured biochemical parameters. Elevated blood urea nitrogen may indicate impaired renal function, reduced effective circulating volume, or increased catabolism, whereas decreased albumin often reflects poor nutritional reserve, greater inflammatory burden, and reduced physiologic reserve. By combining these two parameters into a single ratio, BAR may provide a more comprehensive reflection of the systemic condition of older patients after HF. Previous studies have shown that BAR is associated with in hospital mortality in older emergency department patients, prognosis in patients with sepsis, and short term outcomes in patients with heart failure (18–20). Nevertheless, the prognostic value of BAR in older patients with HF remains insufficiently studied.

Therefore, using data from a retrospective cohort of surgically treated patients aged 60 years or older with HF, this study systematically compared multiple inflammation and nutrition related composite indicators in relation to the risk of 1-year all-cause mortality after HF, with a particular focus on the independent association, dose response relationship, predictive performance, clinical net benefit, and subgroup consistency of BAR.

## 2 Materials and methods

### 2.1 Study design

This was a single center, retrospective cohort study. We consecutively screened patients who were hospitalized for HF and underwent surgical treatment at a regional tertiary trauma center in eastern China between May 2016 and December 2021. Clinical data and laboratory test results were obtained from the electronic medical record system. Survival status at 1-year after HF was ascertained through electronic medical records and telephone follow up.

This study was conducted in accordance with the principles of the Declaration of Helsinki and was reported according to the STROBE guidelines. The study protocol was approved by the ethics review committee of the study hospital, with the approval number KYXM-202212-017. Because this was a retrospective study and all data were anonymized before analysis, the requirement for written informed consent was waived by the ethics committee.

### 2.2 Study population

The inclusion criteria were as follows: (1) age 60 years or older; (2) a confirmed diagnosis of femoral neck fracture or intertrochanteric fracture; (3) receipt of surgical treatment during hospitalization; (4) availability of clinical records and laboratory test results within 24 h after admission; and (5) ascertainable survival status at 1 year after hip fracture.

The exclusion criteria were as follows: (1) age younger than 60 years; (2) pathological fracture; (3) multiple trauma; (4) malignant tumor, acute cardiovascular or cerebrovascular disease, or other major conditions that could substantially affect 1-year prognosis; (5) long term bedridden status before fracture; (6) no surgical treatment; (7) missing survival follow-up data; and (8) Variable missing proportion ≥ 20%.

No formal sample size calculation was performed before the study. The sample size was determined by the number of patients who met the inclusion criteria and did not meet any exclusion criteria during the study period.

Data extraction and follow up were independently performed by 2 investigators, and data accuracy was checked by a third independent investigator. For discrepant or inconsistent records, the final data were determined after review of the original electronic medical records by the research team.

### 2.3 Variables and outcome

All baseline variables included in the analysis were extracted from clinical records after admission or from the first laboratory test results obtained within 24 h after admission. The collected data included demographic characteristics, fracture related information, comorbidities, laboratory indicators, and perioperative information.

Demographic and fracture related variables included sex, age, fracture side, fracture to admission time (F_time), fracture type, and surgical duration (S_time). F_time was defined as the time from fracture occurrence to hospital admission and was measured in days. S_time was defined as the time from the start to the end of the surgical procedure and was measured in minutes. Comorbidities included hypertension, coronary heart disease (CHD), diabetes mellitus (DM), cerebral infarction (CI), and chronic bronchitis (CB). Laboratory indicators included complete blood count, biochemical tests, and coagulation parameters. The complete list of variables is shown in Table 1.

**TABLE 1:** Characteristics of all enrolled patients.

Variables	Total (*n* = 521)	Survivors (*n* = 444)	Non-survivors (*n* = 77)	*P*
Sex (female)	336 (64)	293 (66)	43 (56)	0.112
Age (year)	78 (71, 84)	78 (71, 84)	83 (77, 87)	<0.001
HBP (yes)	260 (50)	225 (51)	35 (45)	0.47
CHD (yes)	96 (18)	71 (16)	25 (32)	0.001
DM (yes)	95 (18)	76 (17)	19 (25)	0.154
CI (yes)	129 (25)	101 (23)	28 (36)	0.016
CB (yes)	52 (10)	39 (9)	13 (17)	0.047
F_side (left)	265 (51)	225 (51)	40 (52)	0.934
F_time (day)	1 (1, 2)	1 (1, 2)	1 (1, 3)	0.115
F_type (FNF)	284 (55)	246 (55)	38 (49)	0.389
S_time (min)	70 (55, 85)	70 (55, 85)	70 (60, 80)	0.456
WBC (10^9^/L)	7.71 (6.29, 9.38)	7.67 (6.29, 9.25)	8.24 (6.33, 9.82)	0.208
N (10^9^/L)	5.8 (4.62, 7.33)	5.8 (4.59, 7.19)	6.16 (4.83, 8.35)	0.186
L (10^9^/L)	0.99 (0.76, 1.31)	0.99 (0.77, 1.32)	0.96 (0.72, 1.26)	0.291
M (10^9^/L)	0.57 (0.44, 0.75)	0.56 (0.44, 0.73)	0.6 (0.48, 0.83)	0.078
RBC (10^12^/L)	3.6 (3.15, 4)	3.63 (3.21, 4.02)	3.36 (3.01, 3.82)	0.004
HB (g/L)	109 (94, 120)	110 (95.75, 121)	98 (86, 113)	0.002
PLT (10^9^/L)	151 (113, 196)	151 (112.5, 194.25)	150 (116, 208)	0.653
GLU (mmol/L)	6.1 (5.4, 7.2)	6 (5.4, 7.03)	6.5 (5.4, 7.6)	0.107
ALT (u/L)	16 (12, 22)	15 (12, 22)	18 (12, 24)	0.316
AST (u/L)	22 (18, 27)	21 (18, 26)	23 (18, 29)	0.206
DBIL (μmol/L)	6 (4.6, 8.8)	6.2 (4.8, 8.5)	5.6 (4.3, 9.4)	0.61
IBIL (μmol/L)	11 (8, 14)	11 (8.17, 14.3)	9 (6.2, 13)	0.003
ALB (g/L)	37.7 (34.3, 40)	38 (34.68, 40.23)	36.3 (32, 38.7)	<0.001
GLOB (g/L)	28.14 ± 4.65	27.99 ± 4.61	29.04 ± 4.83	0.081
BUN (mmol/L)	6.7 (5.4, 8.6)	6.5 (5.3, 8.3)	8.4 (6.6, 11.3)	<0.001
Cr (umol/L)	66 (55, 84)	65 (54, 81)	75 (58, 119)	<0.001
CysC (mg/L)	1.09 (0.87, 1.36)	1.06 (0.86, 1.31)	1.28 (1.02, 1.79)	<0.001
Ka (mmol/L)	3.78 (3.5, 4.12)	3.75 (3.5, 4.1)	3.95 (3.52, 4.26)	0.052
Na (mmol/L)	140 (138, 142)	140 (138, 142)	140 (138, 141)	0.223
Ca (mmol/L)	2.13 (2.06, 2.2)	2.13 (2.07, 2.2)	2.1 (2.03, 2.18)	0.126
PT (s)	13.3 (12.7, 13.9)	13.3 (12.7, 13.9)	13.6 (13, 14.2)	0.021
INR	1.01 (0.96, 1.08)	1.01 (0.95, 1.07)	1.04 (0.98, 1.11)	0.048
PTA (%)	98 (89, 108)	98 (90, 109)	94 (85, 104)	0.043
APTT (s)	36.9 (34, 40.2)	36.7 (33.9, 40.1)	38.3 (34.3, 41.5)	0.061
TT (s)	16.8 (16, 17.7)	16.8 (16.1, 17.6)	16.6 (15.9, 17.9)	0.911
FIB (g/L)	3.78 (3.17, 4.64)	3.76 (3.16, 4.63)	4.17 (3.35, 4.86)	0.058

HBP, hypertension; CHD, coronary heart disease; DM, diabetes mellitus; CI, cerebral infarction; CB, chronic bronchitis; F_side, fracture side; F_time, fracture-to-admission time; F_type, fracture type; FNF, femoral neck fracture; S_time, surgical duration; WBC, white blood cell count; N, neutrophil count; L, lymphocyte count; M, monocyte count; RBC, red blood cell count; HB, hemoglobin; PLT, platelet count; GLU, glucose; ALT, alanine aminotransferase; AST, aspartate aminotransferase; DBIL, direct bilirubin; IBIL, indirect bilirubin; ALB, albumin; GLOB, globulin; BUN, blood urea nitrogen; Cr, creatinine; CysC, cystatin C; Ka, potassium; Na, sodium; Ca, calcium; PT, prothrombin time; INR, international normalized ratio; PTA, prothrombin activity; APTT, activated partial thromboplastin time; TT, thrombin time; FIB, fibrinogen.

The primary outcome was 1-year all-cause mortality after HF, defined as death from any cause within 1-year after fracture occurrence. Survival status was first determined from discharge records, outpatient and emergency department records, readmission records, and death records in the electronic medical record system. For patients whose 1-year survival status after HF could not be clearly determined from electronic medical records, telephone follow up was conducted using the contact information of the patient or family members recorded in the medical record system. Patients whose 1-year mortality outcome could not be confirmed were excluded.

### 2.4 Composite indicators

Inflammation and nutrition related composite indicators were calculated from complete blood count and biochemical test results. These indicators included the neutrophil to lymphocyte ratio (NLR), monocyte to lymphocyte ratio (MLR), platelet to lymphocyte ratio (PLR), systemic immune inflammation index (SII), systemic inflammation response index (SIRI), aggregate index of systemic inflammation (AISI), HALP index, prognostic nutritional index (PNI), blood urea nitrogen to albumin ratio (BAR), and neutrophil percentage to albumin ratio (NPAR).

This study focused on the association between BAR and 1-year all-cause mortality after HF. BAR was defined as the ratio of blood urea nitrogen (BUN) to albumin (ALB), calculated as follows:


BAR=[BUN⁢(mmol/L)×2.8×10]/ALB⁢(g/L)

All composite indicators were calculated after standardization of measurement units. The detailed formulas and unit conversion rules for each composite indicator are provided in Supplementary Material 1.

### 2.5 Missing data

Missing data were first evaluated, and the distribution and pattern of missingness were visualized using the “VIM” package. According to the prespecified exclusion criteria, patients with missingness of 20% or greater among the variables included in the analysis were excluded. For the remaining missing values in the final analytic dataset, multiple imputation was performed using the “mice” package. The imputed dataset was used for subsequent statistical analyses.

### 2.6 Statistical analysis

The distribution of continuous variables was first assessed for normality using the Shapiro-Wilk test and histograms with theoretical normal distribution curves. Continuous variables with a normal distribution were expressed as mean ± standard deviation and compared using the independent samples *t*-test. Continuous variables with a non-normal distribution were expressed as median and interquartile range and compared using the Wilcoxon rank sum test. Categorical variables were expressed as counts and percentages and compared using the χ^2^-test or Fisher exact test, as appropriate. Baseline characteristics between the 1-year mortality group and the survival group were compared using the “CBCgrps” package.

Logistic regression models were used to evaluate the associations between each composite indicator and the risk of 1-year all-cause mortality after HF. Effect estimates were reported as odds ratios (ORs) with 95% confidence intervals (CIs). First, univariate logistic regression was performed for all candidate clinical variables, laboratory indicators, and composite indicators to identify factors potentially associated with 1-year all-cause mortality after hip fracture. Composite indicators with *P* < 0.05 in univariate analysis were further evaluated using multivariable logistic regression models to assess their independent associations with 1-year all-cause mortality.

To reduce potential multicollinearity, correlation analysis was performed for clinical variables, laboratory indicators, and composite indicators with *P* < 0.05 in univariate logistic regression. Correlation analysis was conducted using the “corrplot” package. A larger absolute correlation coefficient indicated a stronger correlation. Correlation coefficients of 0.40–0.60, 0.60–0.80, and 0.80–1.00 were considered to indicate moderate, strong, and very strong correlations, respectively. Single variables that showed a moderate or stronger correlation with the target composite indicator, defined as *r* > 0.40, were generally not included in the same multivariable model to reduce the potential influence of collinearity.

For each eligible composite indicator, 3 logistic regression models were constructed. Model 1 was unadjusted. Model 2 was adjusted for sex, age, fracture side, F_time, fracture type, and S_time. Model 3 was further adjusted for candidate covariates selected on the basis of univariate logistic regression and correlation analysis. For each multivariable model, the variance inflation factor (VIF) was calculated to assess multicollinearity, with VIF > 5 considered to indicate substantial multicollinearity.

For composite indicators that remained consistently associated with 1-year all-cause mortality after HF across different adjusted models, restricted cubic spline (RCS) analysis was further performed to evaluate the dose response relationship. Predictive performance was assessed using receiver operating characteristic (ROC) curves, and the area under the curve (AUC) was calculated. To further assess the robustness of the predictive performance of BAR, a perturbation-based sensitivity analysis was conducted. Specifically, a 10% random perturbation was applied to BAR, followed by 40-repeated rounds of 10-fold cross validation, to examine the distribution and variation of the AUC under mild data fluctuation and thereby evaluate the stability of the predictive performance of BAR. For the composite indicator with the best predictive performance, subgroup analyses were further performed according to sex, hypertension, CHD, DM, CI, CB, fracture side, and fracture type to assess the robustness of the findings.

All statistical analyses were performed using R software, version 4.4.1. All tests were 2 sided, and *P* < 0.05 was considered statistically significant.

## 3 Results

### 3.1 Study population

**FIGURE 1:**
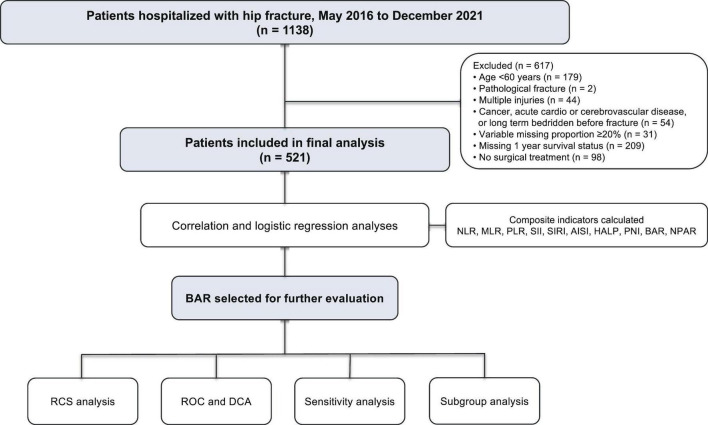
Flowchart of data screening and analysis. AISI, aggregate index of systemic inflammation; BAR, blood urea nitrogen to albumin ratio; DCA, decision curve analysis; HALP, hemoglobin, albumin, lymphocyte, and platelet index; MLR, monocyte to lymphocyte ratio; NLR, neutrophil to lymphocyte ratio; NPAR, neutrophil percentage to albumin ratio; PLR, platelet to lymphocyte ratio; PNI, prognostic nutritional index; RCS, restricted cubic spline; ROC, receiver operating characteristic; SII, systemic immune inflammation index; SIRI, systemic inflammation response index.

A total of 521 patients aged 60 years or older who underwent surgery for HF were included in the final analysis. The patient screening and analytic flow are shown in Figure 1. Among these patients, 444 survived and 77 died within 1 year after HF, corresponding to a 1-year all-cause mortality rate of 14.8%.

According to the prespecified criteria, patients with missingness of 20% or greater among the variables included in the analysis were excluded during patient screening. Evaluation and visualization of missing data in the final analytic cohort showed that a small amount of missingness remained, but the missing proportion for each variable was less than 1% (Supplementary Material 2). Missing values in the analytic dataset were handled using multiple imputation. Assessment of the distribution of continuous variables showed that, except for GLOB, all continuous variables were not normally distributed. Histograms with theoretical normal distribution curves further supported these findings (Supplementary Material 3). Therefore, unless otherwise specified, continuous variables are presented as medians and interquartile ranges, and categorical variables are presented as counts and percentages.

### 3.2 Baseline characteristics

With regard to clinical characteristics, patients who died within 1 year were older than survivors [83 (77, 87) years vs. 78 (71, 84) years, *P* < 0.001] and had higher proportions of CHD (32% vs. 16%, *P* = 0.001), CI (36% vs. 23%, *P* = 0.016), and CB (17% vs. 9%, *P* = 0.047). No statistically significant differences were observed between the 2 groups in sex, fracture side, fracture type, fracture to admission time, or surgical duration.

For laboratory findings, patients who died had lower red blood cell counts (RBC) [3.36 (3.01, 3.82) × 10^12^/L vs. 3.63 (3.21, 4.02) × 10^12^/L, *P* = 0.004], lower hemoglobin (HB) levels [98 (86, 113) g/L vs. 110 (95.75, 121) g/L, *P* = 0.002], lower indirect bilirubin (IBIL) levels [9.0 (6.2, 13.0) μmol/L vs. 11.0 (8.17, 14.3) μmol/L, *P* = 0.003], and lower albumin (ALB) levels [36.3 (32.0, 38.7) g/L vs. 38.0 (34.68, 40.23) g/L, *P* < 0.001]. In contrast, patients who died had higher BUN levels [8.4 (6.6, 11.3) mmol/L vs. 6.5 (5.3, 8.3) mmol/L, *P* < 0.001], higher Cr levels [75 (58, 119) μmol/L vs. 65 (54, 81) μmol/L, *P* < 0.001], and higher CysC levels [1.28 (1.02, 1.79) mg/L vs. 1.06 (0.86, 1.31) mg/L, *P* < 0.001]. For coagulation parameters, patients who died had longer PT [13.6 (13.0, 14.2) s vs. 13.3 (12.7, 13.9) s, *P* = 0.021], higher INR [1.04 (0.98, 1.11) vs. 1.01 (0.95, 1.07), *P* = 0.048], and lower PTA [94 (85, 104) vs. 98 (90, 109), *P* = 0.043]. Overall, patients who died showed greater age, higher comorbidity burden, anemia, poorer nutritional status, renal dysfunction, and coagulation abnormalities at admission. The complete baseline characteristics of the two groups are shown in Table 1.

### 3.3 Regression analysis

Univariate logistic regression analysis showed that age, CHD, CI, CB, RBC, HB, GLU, IBIL, ALB, BUN, Cr, and CysC were associated with 1-year all-cause mortality after HF. Among the composite indicators, SIRI, AISI, HALP, PNI, BAR, and NPAR were associated with 1-year all-cause mortality (Supplementary Material 4).

**FIGURE 2:**
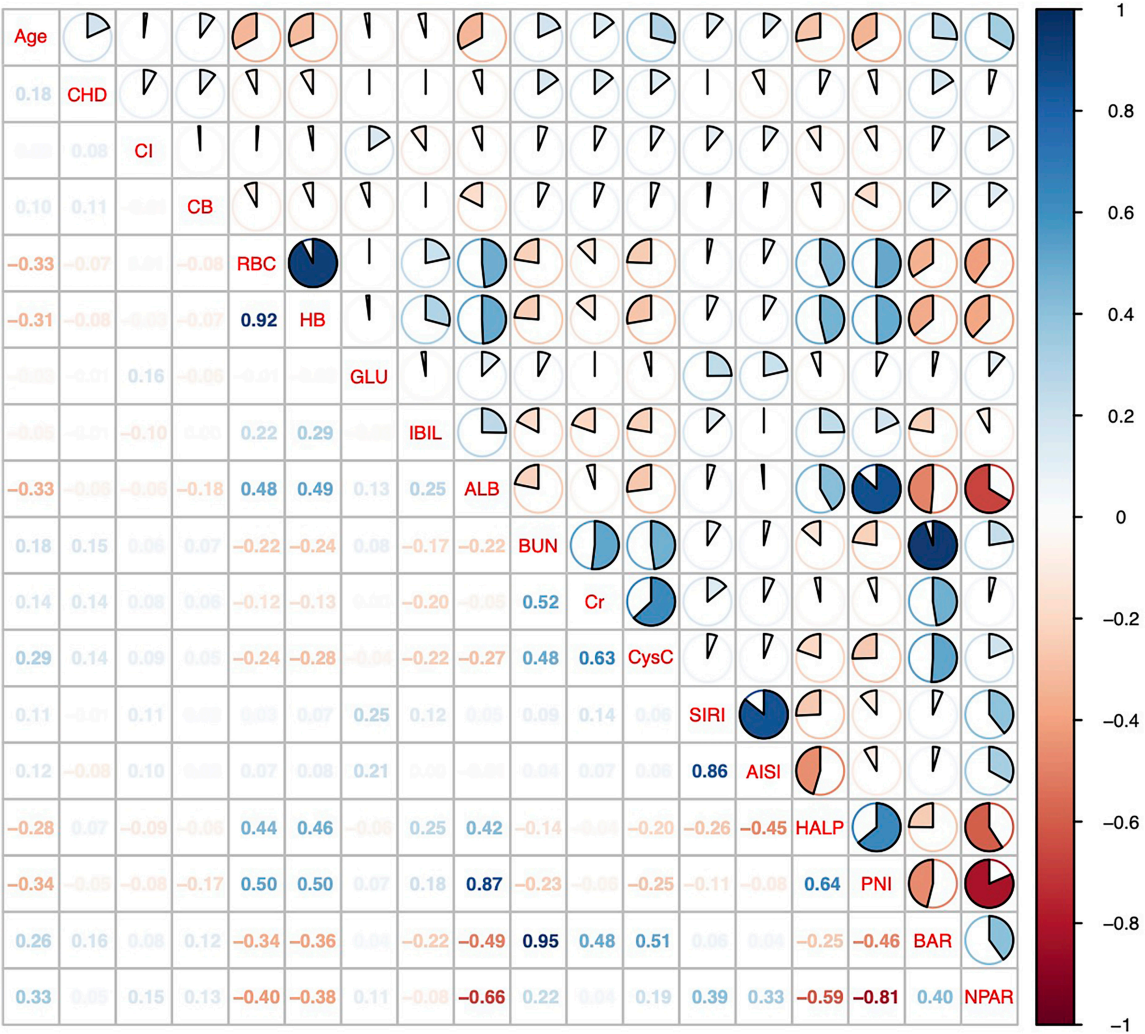
Heatmap of correlation coefficients between variables.

Further correlation analysis showed moderate or stronger correlations between several composite indicators and candidate clinical or laboratory variables. HALP was correlated with RBC, HB, and ALB; PNI was correlated with RBC, HB, and ALB; BAR was correlated with ALB, BUN, Cr, and CysC; and NPAR was correlated with RBC and ALB (Figure 2). After excluding single variables that showed moderate or stronger correlations with the target composite indicators, age, coronary heart disease, cerebral infarction, chronic bronchitis, GLU, and IBIL were included as candidate adjustment variables in model 3.

For composite indicators that were statistically significant in univariate analysis, 3 logistic regression models were constructed. Model 1 was unadjusted. Model 2 was adjusted for sex, age, F_side, F_time, F_type, and S_time. Model 3 was further adjusted for CHD, CI, CB, GLU, and IBIL. SIRI, AISI, HALP, PNI, and NPAR were associated with mortality risk in the unadjusted model or in partially adjusted models, but these associations were attenuated or no longer statistically significant after further adjustment.

**FIGURE 3:**
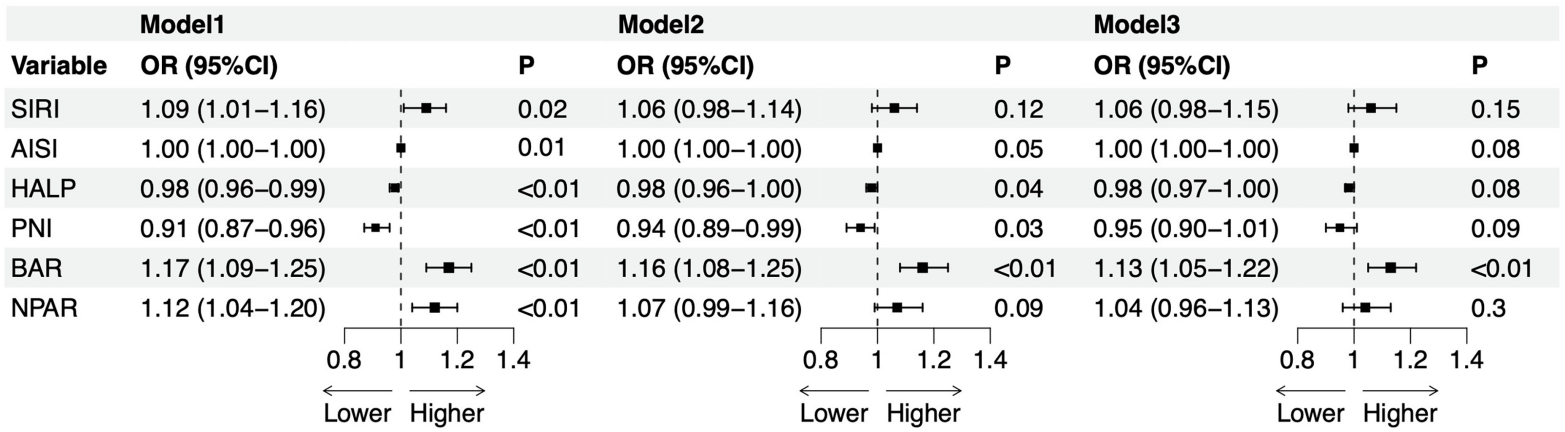
Forest plot of logistic regression model.

In contrast, only BAR remained consistently associated with 1-year all-cause mortality after HF across all 3 models. The ORs for BAR in models 1, 2, and 3 were 1.17 (95% CI: 1.09–1.25, *P* < 0.01), 1.16 (95% CI: 1.08–1.25, *P* < 0.01), and 1.13 (95% CI: 1.05–1.22, *P* < 0.01), respectively (Figure 3). The VIF values for all variables in the multivariable models were less than 5, suggesting no substantial multicollinearity.

### 3.4 Dose response and predictive performance

**FIGURE 4:**
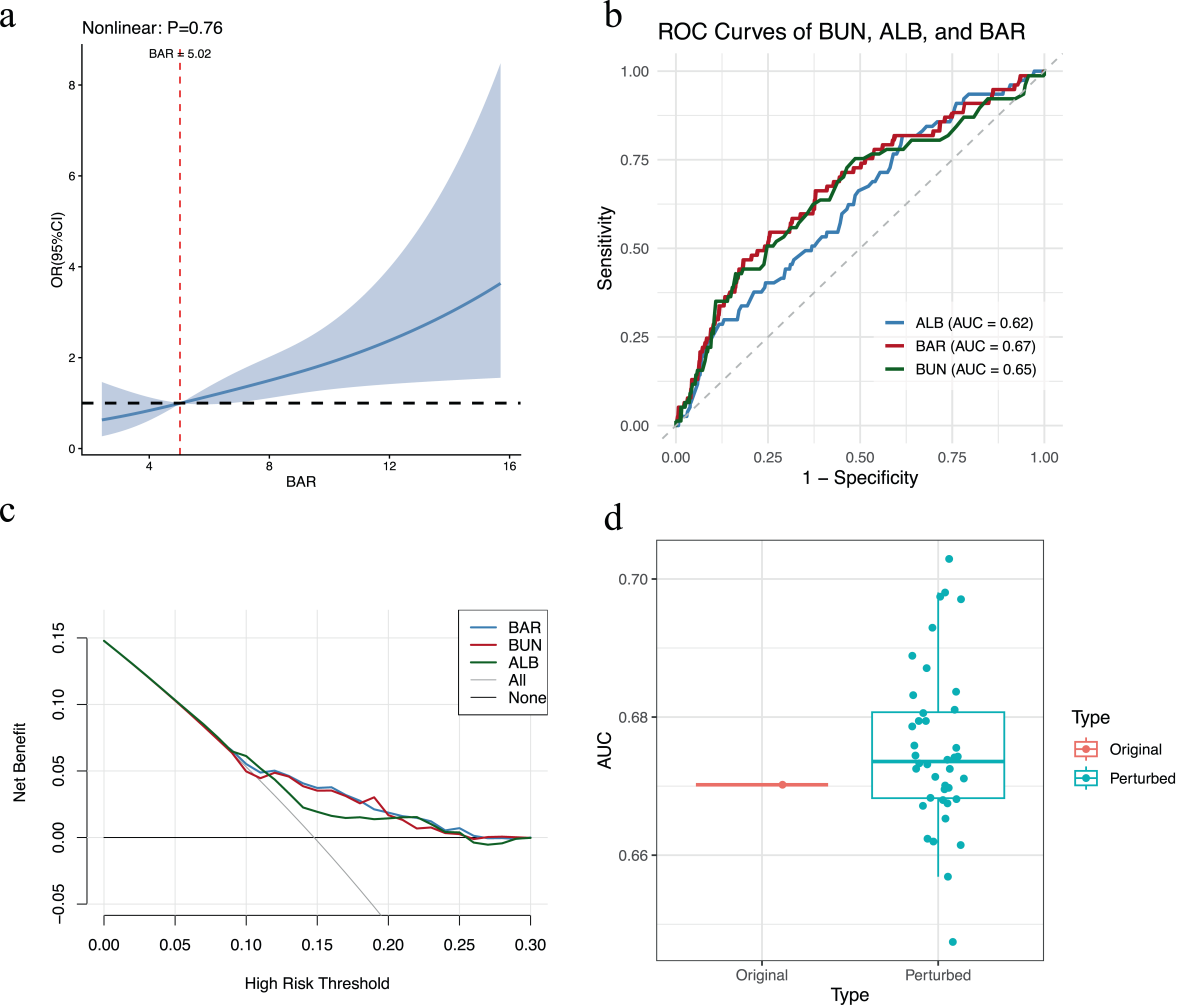
Prognostic performance and sensitivity analysis of BAR. **(a)** Restricted cubic spline analysis showing the association between BAR and 1-year mortality. **(b)** Receiver operating characteristic (ROC) curves of BAR, BUN, and ALB. **(c)** Decision curve analysis (DCA) comparing the clinical net benefit of BAR, BUN, and ALB. **(d)** Sensitivity analysis of the BAR model.

Because BAR remained consistently associated with 1-year all-cause mortality after HF in different adjusted models, restricted cubic spline analysis was further performed to examine the dose response relationship between BAR and mortality risk. No significant nonlinear association was observed between BAR and 1-year all-cause mortality after HF (P for nonlinearity = 0.76, Figure 4a). The overall trend showed a gradual increase in mortality risk with increasing BAR levels, suggesting that the association between BAR and mortality risk may be approximately linear.

To assess the discriminatory ability of BAR for 1-year all-cause mortality after HF, ROC curves were plotted and AUCs were calculated. The AUC of BAR was 0.67, which was higher than that of BUN (0.65) and ALB (0.62) (Figure 4b). These findings suggest that BAR had modest discriminatory ability for 1-year all-cause mortality and performed better than either BUN or ALB alone.

Decision curve analysis was further used to evaluate the potential net benefit of BAR, BUN, and ALB for clinical risk stratification. Within the threshold probability range shown in the figure, BAR, BUN, and ALB all showed some net benefit and generally outperformed the strategies of treating all patients or treating no patients. The BAR curve was above the BUN and ALB curves across most threshold ranges, suggesting that BAR may provide relatively greater value for clinical risk stratification and auxiliary decision making (Figure 4c).

In addition, a perturbation-based sensitivity analysis was performed to evaluate the robustness of the predictive performance of BAR. After applying a 10% perturbation to BAR and repeating 40 rounds of 10-fold cross validation, the distribution of AUCs under the perturbed data was close to the original AUC, with a narrow overall range and no obvious decline. These findings suggest that BAR maintained relatively stable performance under mild data perturbation (Figure 4d).

### 3.5 Subgroup analysis

**FIGURE 5:**
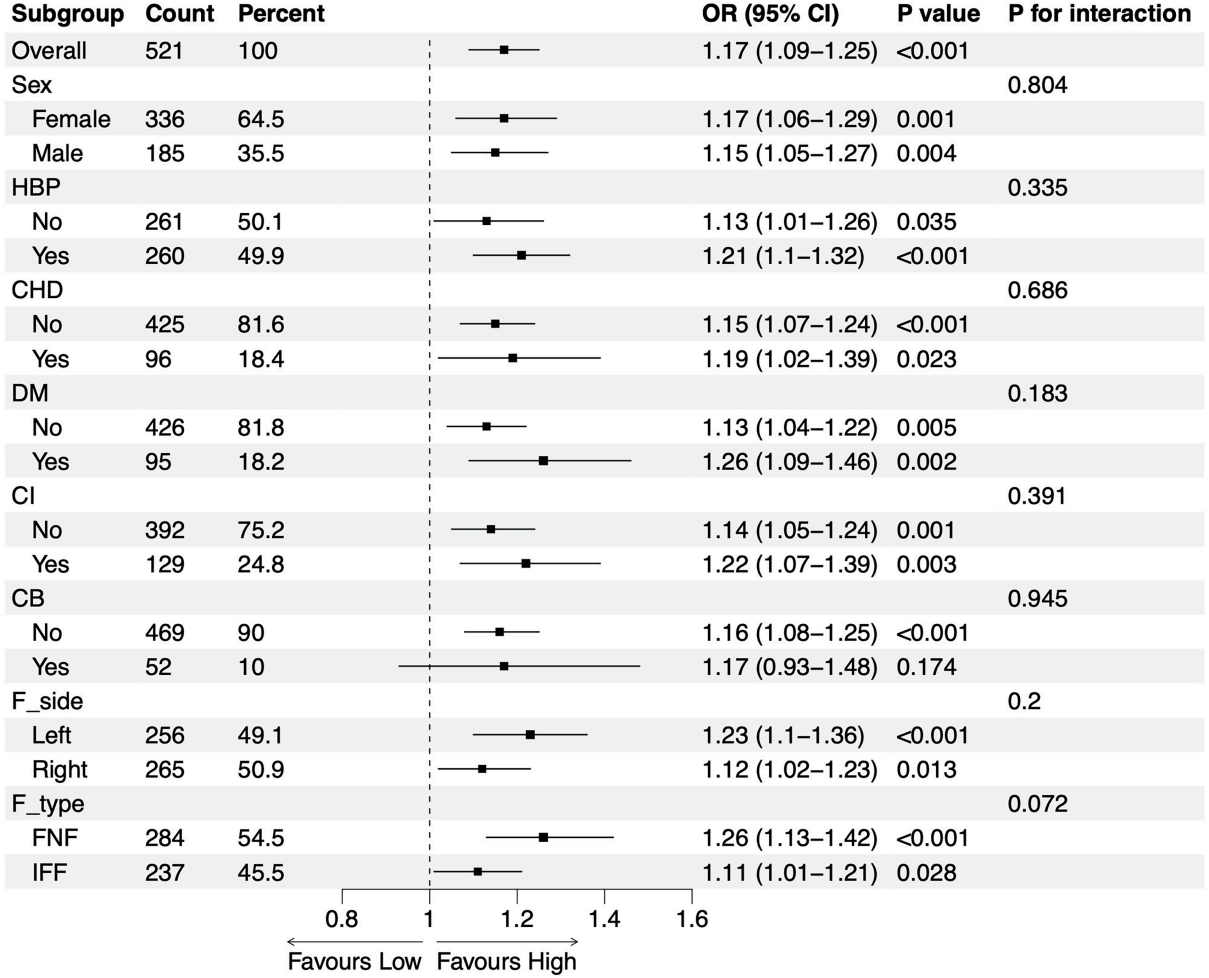
Forest plot of subgroup analysis.

To further assess the consistency of the association between BAR and 1-year all-cause mortality after HF across clinical subgroups, subgroup analyses were performed according to sex, hypertension, CHD, DM, CI, CB, fracture side, and fracture type. Except for the chronic bronchitis subgroup, higher BAR levels were generally associated with a higher mortality risk across subgroups. All *P*-values for interaction were greater than 0.05, suggesting that the association between BAR and 1-year all-cause mortality after HF was not significantly modified by sex, major comorbidities, fracture side, or fracture type (Figure 5).

## 4 Discussion

In this study, we systematically compared 10 inflammation and nutrition related composite indicators in relation to the risk of 1-year all-cause mortality after HF surgery in patients aged 60 years or older. The main findings were that SIRI, AISI, HALP, PNI, BAR, and NPAR were associated with mortality risk in univariate analysis; however, after further adjustment for demographic characteristics, fracture related factors, surgical duration, and candidate confounders, only BAR remained consistently associated with 1-year all-cause mortality after HF. Restricted cubic spline analysis did not show a significant nonlinear association between BAR and mortality risk, suggesting an approximately linear increase in risk. In ROC analysis, the AUC of BAR was 0.67, which was higher than that of BUN and ALB, indicating that BAR provided modest incremental discriminatory ability compared with its individual components, although its overall predictive performance remained moderate. These findings suggest that BAR may serve as a simple auxiliary indicator derived from routine biochemical testing for early risk identification in older patients with HF.

In this study, patients who died within 1 year already showed a more unfavorable clinical profile at admission, including older age, greater comorbidity burden, anemia, lower albumin levels, higher renal function related markers, and coagulation abnormalities. These findings are consistent with previous studies on adverse outcomes after HF in older adults. Prior cohort studies have shown that long term mortality after HF is not determined solely by fracture type or surgical approach, but is influenced by multiple factors, including age, comorbidities, preoperative physiologic reserve, nutritional status, renal function, anemia, and perioperative complications (7, 21, 22). Laboratory indicators such as hypoalbuminemia, anemia, and renal dysfunction have also been associated with postoperative complications, poor functional recovery, and increased mortality risk (23–25). Therefore, laboratory findings obtained early after admission may reflect not only the physiologic consequences of acute trauma, but also the cumulative burden of chronic disease and systemic frailty before fracture.

In recent years, several composite indicators derived from routine blood tests have been used for prognostic assessment in patients with HF. The association between NLR and postoperative mortality in older patients with HF has been summarized in a meta-analysis (14). Hematologic inflammatory indicators, including SIRI, have also been reported to be associated with mortality risk in older patients with HF (15, 26). Furthermore, Kaya et al. found that older patients undergoing hip fracture surgery had a higher risk of death when their preoperative C-reactive protein-to-albumin ratio (CAR) was elevated. This suggests that both inflammatory burden and nutritional status may contribute to unfavorable outcomes in this population (27). Regarding nutrition related indicators, a systematic review showed that several nutritional indices were associated with mortality after HF (16), and a recent study suggested that PNI could predict longer term mortality in older patients with HF (17). However, in the present study, the associations of SIRI, AISI, HALP, PNI, and NPAR were attenuated or no longer statistically significant after further adjustment, whereas BAR remained consistently associated with mortality across different models. One possible explanation is that inflammatory cell counts and nutritional scores are more susceptible to acute trauma responses, occult infection, fluid status, and short-term stress related fluctuations. In contrast, BAR integrates information on renal function or perfusion status, catabolism, and nutritional reserve, and may more closely capture the systemic pathophysiologic burden underlying long term mortality risk in older patients with HF.

The potential clinical relevance of BAR may arise from the complementary information provided by BUN and ALB. BUN is a nitrogenous end product of protein catabolism and is mainly excreted by the kidney. Its level is influenced by glomerular filtration, effective circulating volume, tissue perfusion, protein catabolism, and disease related stress (28). In older patients with HF, pain, fasting, volume depletion, inflammation, reduced baseline renal reserve, and posttraumatic hypercatabolism may all contribute to higher BUN levels. Previous studies have shown that BUN is associated with adverse outcomes in several clinical conditions, including heart failure, acute pancreatitis, infectious diseases, and stroke (29–31). After trauma, inflammatory mediator release, neuroendocrine activation, and enhanced protein breakdown may further alter nitrogen metabolism (32–34). In addition, preoperative dehydration or volume depletion is not uncommon in patients with HF and may increase BUN through reduced renal blood flow and perfusion (35, 36). Existing evidence also suggests that BUN, Cr, CysC, estimated glomerular filtration rate, and acute kidney injury are associated with adverse outcomes in patients with HF (37–39).

ALB reflects overall patient status from the perspectives of nutritional reserve and inflammatory burden. Albumin is synthesized by the liver and, beyond maintaining colloid osmotic pressure, has antioxidant, anti-inflammatory, endothelial stabilizing, and immunomodulatory properties (40–42). Hypoalbuminemia is commonly seen in malnutrition, chronic wasting, inflammation, and acute illness. Classic surgical studies have shown that low albumin levels are associated with postoperative complications, prolonged hospital stay, and increased mortality (43–45). In acute trauma and inflammatory states, increased capillary permeability, albumin extravasation, impaired hepatic synthesis, and inadequate nutritional intake may all contribute to lower serum albumin levels (46–48). In patients with HF, hypoalbuminemia has been reported to be associated with postoperative complications and increased mortality risk (24, 49, 50). At the same time, malnutrition, sarcopenia, and frailty often overlap in patients with fragility HF and are closely related to functional recovery and long term survival (11, 13).

Accordingly, an elevated BAR may reflect the combined presence of two risk signals. On the one hand, higher BUN may indicate renal dysfunction or impaired perfusion, volume depletion, or increased catabolism. On the other hand, lower ALB may indicate poor nutritional reserve, increased inflammatory burden, or reduced overall physiologic reserve. Compared with BUN or ALB alone, BAR integrates these two dimensions into a single ratio and may better reflect systemic vulnerability after trauma in older patients with HF. Prior studies have shown that BAR is associated with adverse outcomes in various acute and critical illnesses, including in hospital mortality in older emergency department patients, prognosis in sepsis, short term outcomes in heart failure, pneumonia, acute gastrointestinal bleeding, and pulmonary embolism (51–54). By applying BAR to older patients with HF and observing its stable association with 1-year all-cause mortality, the present study suggests that BAR may have potential value for risk identification across disease settings.

In the present study, RCS analysis did not show a significant nonlinear association between BAR and mortality risk, suggesting that the risk of death may increase gradually with increasing BAR levels. For older patients with HF, nutritional reserve, volume status, renal function, and metabolic stress usually vary along a continuum rather than around a single distinct threshold. Therefore, BAR should not be interpreted solely on the basis of a fixed cutoff value, but rather as a continuous risk indicator within a broader clinical assessment. Clinically, patients with higher BAR levels may require more active evaluation of volume status, renal reserve, malnutrition, hypoalbuminemia, anemia, and inflammatory burden, as well as greater attention to perioperative monitoring, nutritional support, and orthogeriatric co management.

It should be emphasized that the stable association between BAR and mortality does not mean that BAR can independently predict prognosis. In this study, the AUC of BAR was 0.67, indicating moderate discriminatory ability. Although this was higher than the AUCs of BUN and ALB, the improvement was limited. BAR should therefore be regarded as an auxiliary signal for early risk identification rather than a substitute for comprehensive clinical judgment or a complete predictive model. Indeed, a previous systematic review showed that mortality prediction models for HF vary substantially in terms of included variables, validation quality, and clinical applicability, and no single model or indicator can fully meet the need for risk stratification across different clinical settings (10). The advantage of BAR lies not in complex modeling or maximal predictive performance, but in its availability from routine biochemical testing, ease of calculation, low cost, and interpretability. When combined with age, comorbidities, anemia, renal function, coagulation status, nutritional assessment, frailty assessment, and perioperative management strategies, BAR may help improve the efficiency of early identification of high-risk patients after admission.

From a clinical perspective, elevated BAR may serve as a signal that a patient requires more comprehensive perioperative assessment. Older patients with HF often need timely surgical risk evaluation, medical optimization, and perioperative planning. If BAR is elevated at admission, clinicians may more actively assess for volume depletion, reduced renal reserve, hypoalbuminemia, malnutrition, or potential complication risk, and may consider closer postoperative monitoring, nutritional support, and multidisciplinary co management. Previous studies have shown that orthogeriatric co management can improve process quality and is associated with lower mortality risk and better clinical outcomes in some patient populations (55–57). Therefore, BAR should be positioned as a low-cost risk alert indicator rather than an independent decision-making tool.

This study has several strengths. First, we compared multiple commonly used inflammation and nutrition related composite indicators within the same cohort, rather than focusing on a single indicator, which helped clarify the relative value of BAR among different composite measures. Second, correlation analysis and VIF assessment were performed during model construction to reduce the potential influence of collinearity between composite indicators and their component variables or related laboratory indicators. Third, in addition to logistic regression, we used RCS analysis, ROC analysis, sensitivity analysis, and subgroup analysis to evaluate BAR from multiple perspectives, including association strength, dose response, discrimination, and internal robustness. Fourth, BAR was calculated from routine biochemical tests obtained early after admission, which reflects real world clinical practice and supports its potential feasibility.

This study also has limitations. First, as a single center retrospective cohort study, it may be affected by selection bias, information bias, and residual confounding, although consecutive screening, independent data extraction, multiple imputation, and multivariable adjustment were used. Second, the study included only patients who underwent surgery and had ascertainable 1-year survival status, while patients with pathological fractures, multiple trauma, prefracture long term bedridden status, malignant tumors, and acute major illnesses were excluded. Therefore, the findings cannot be directly generalized to all patients with HF. Third, several important prognostic factors were not included in the analysis, including ASA classification, cognitive function, frailty score, prefracture mobility, anesthesia type, specific surgical procedure, time from admission to surgery, transfusion, postoperative complications, rehabilitation intensity, nutritional intervention, and anti-osteoporosis treatment. Among these factors, time to surgery is an important indicator of perioperative management in older patients with HF, and current evidence supports early surgery within 24 h when clinically feasible (58). The absence of these important prognostic factors may have introduced residual confounding and should be considered when interpreting our findings. Future prospective studies should systematically collect relevant perioperative variables to further validate the present results. Fourth, only a single BAR value early after admission was evaluated, and dynamic changes in BAR during the perioperative and rehabilitation periods were not analyzed. Early-admission BAR may reflect the patient’s baseline status at presentation and may be useful for early risk assessment. However, older patients with hip fracture often experience clinical changes during hospitalization. Surgery, blood transfusion, infection, nutritional support, fluid management, and rehabilitation interventions may all affect subsequent BUN and albumin levels. Therefore, BAR measured at a single time point may not fully capture the dynamic evolution of the patient’s clinical condition. Future studies should adopt a longitudinal design to further evaluate changes in BAR over time and their association with clinical outcomes. Fifth, the outcome was all cause mortality, and specific causes of death, such as cardiovascular events, infection, renal failure, or other causes, could not be distinguished. Sixth, sensitivity and subgroup analyses can provide only evidence of internal consistency and cannot replace independent external validation. Given potential differences across regions in patient characteristics, perioperative management, rehabilitation resources, and long-term care conditions, multicenter prospective studies are needed to further validate the external applicability of BAR. Finally, it should be noted that the AUC of BAR in this study was 0.67, indicating only moderate discriminative performance. Thus, although our findings support a robust association between BAR and adverse outcomes in older patients with hip fracture, BAR should not be used as a stand-alone tool for clinical decision-making. A more appropriate role for BAR may be as an adjunctive marker, helping to provide an early signal of increased risk at the time of admission. Future prospective studies could further examine whether combining BAR with frailty indices, ASA classification, nutritional assessment, comorbidity indices, and preoperative functional status improves the identification of high-risk patients and helps inform perioperative management, nutritional support, and postoperative rehabilitation planning.

## 5 Conclusion

In conclusion, this study found that BAR was consistently associated with the risk of 1-year all-cause mortality among patients aged 60 years or older who underwent surgery for HF, with generally consistent findings across adjusted models, dose response analysis, sensitivity analysis, and subgroup analysis. By integrating information on renal function and perfusion status, metabolic stress, and nutritional reserve, BAR may provide a simple, low cost, and readily available auxiliary indicator for early risk stratification in older patients with HF. However, its predictive performance was moderate and should not replace comprehensive clinical judgment. Future multicenter, large scale, prospective studies are needed to validate the external applicability of BAR and to evaluate its incremental value when combined with frailty assessment, nutritional assessment, and existing clinical risk models.

## Data Availability

The data analyzed in this study is subject to the following licenses/restrictions: The data supporting the findings of this study are available from the corresponding author upon reasonable request. Requests to access these datasets should be directed to Jiale Guo, jialeguo1997@163.com.
