# Supplementary material for: Blood urea nitrogen to albumin ratio and 1-year mortality after hip fracture surgery in older adults: a retrospective cohort study
Source: Front Med (Lausanne). 2026 Jul 17;13:1877434. doi: 10.3389/fmed.2026.1877434 (PMC13423672; doi:10.3389/fmed.2026.1877434)
Supplement: Supplementary file 1 [file Supplementary_file_1.docx]

Supplementary Material 1. Definitions and Formulas of Composite Indices

| No. | Composite index | Abbreviation | Formula |
| --- | --- | --- | --- |
| 1 | Neutrophil-to-lymphocyte ratio | NLR | NLR = N (10⁹/L) / L (10⁹/L) |
| 2 | Monocyte-to-lymphocyte ratio | MLR | MLR = M (10⁹/L) / L (10⁹/L) |
| 3 | Platelet-to-lymphocyte ratio | PLR | PLR = PLT (10⁹/L) / L (10⁹/L) |
| 4 | Systemic immune-inflammation index | SII | SII = PLT (10⁹/L) × NLR |
| 5 | Systemic inflammation response index | SIRI | SIRI = M (10⁹/L) × NLR |
| 6 | Aggregate index of systemic inflammation | AISI | AISI = N (10⁹/L) × PLT (10⁹/L) × M (10⁹/L) / L (10⁹/L) |
| 7 | Hemoglobin, albumin, lymphocyte, and platelet index | HALP | HALP = [HB (g/L) × ALB (g/L) × L (10⁹/L)] / PLT (10⁹/L) |
| 8 | Prognostic nutritional index | PNI | PNI = ALB (g/L) + 5 × L (10⁹/L) |
| 9 | Blood urea nitrogen-to-albumin ratio | BAR | BAR = [BUN（mmol/L） × 2.8 × 10] / ALB（g/L） |
| 10 | Neutrophil percentage-to-albumin ratio | NPAR | NPAR = neutrophil percentage (%) × 100 / ALB (g/dL) |

Supplementary material 2. Histogram of missing data.


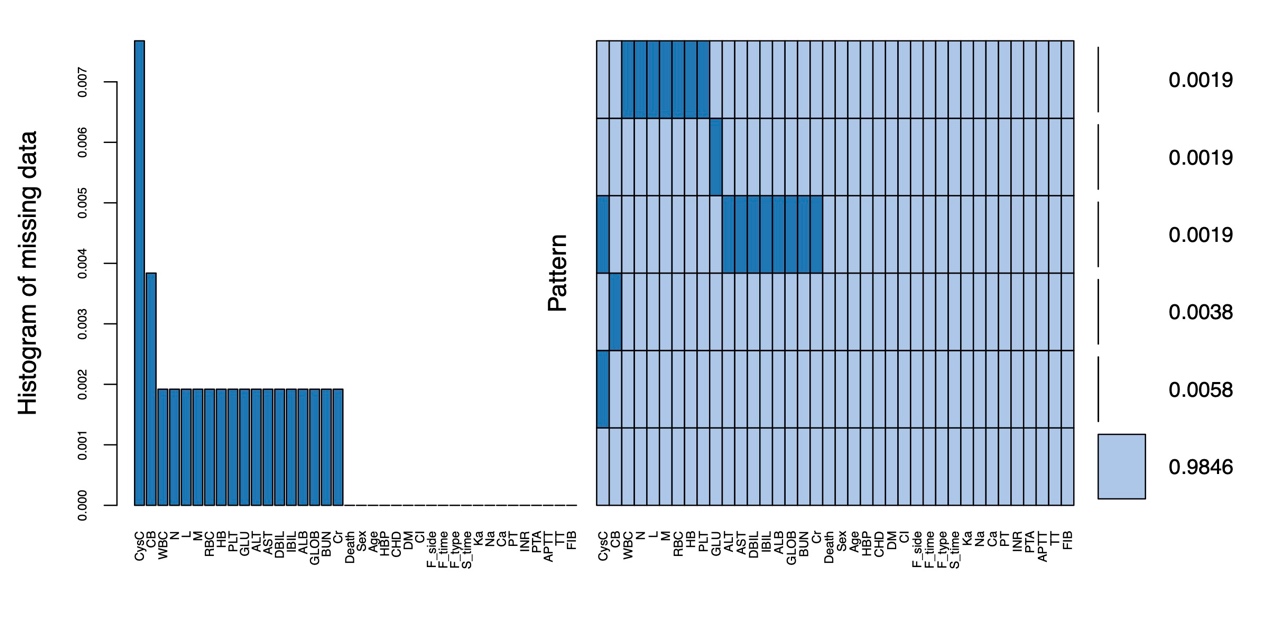


Supplementary material 3. Histogram of the baseline data distribution characteristics.


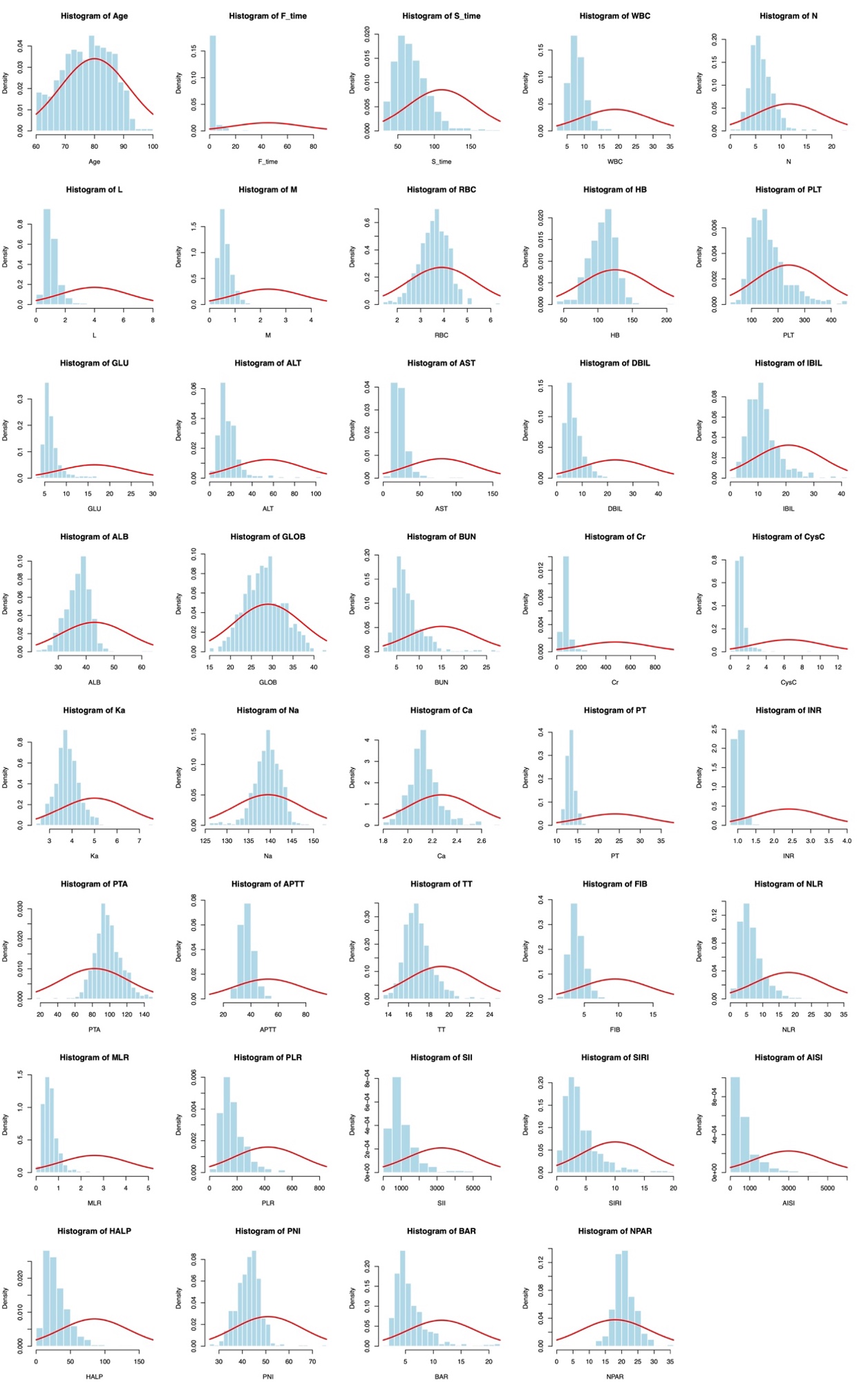


Supplementary material 4. Results of single-factor Logistic regression.

| Variable | OR (95% CI) | P value |
| --- | --- | --- |
| Sex | 1.5343 (0.9392–2.5064) | 0.0874 |
| Age | 1.0762 (1.0419–1.1116) | <0.0001 |
| HBP | 0.8111 (0.4991–1.3182) | 0.3982 |
| CHD | 2.5257 (1.4714–4.3355) | 0.0008 |
| DM | 1.5862 (0.8935–2.8159) | 0.1151 |
| CI | 1.9406 (1.1600–3.2466) | 0.0116 |
| CB | 2.1094 (1.0678–4.1670) | 0.0317 |
| F_side | 1.0523 (0.6484–1.7077) | 0.8367 |
| F_time | 0.9943 (0.9458–1.0454) | 0.8240 |
| F_type | 1.2751 (0.7856–2.0697) | 0.3254 |
| S_time | 0.9966 (0.9860–1.0073) | 0.5360 |
| WBC | 1.0506 (0.9667–1.1416) | 0.2451 |
| N | 1.0730 (0.9728–1.1836) | 0.1587 |
| L | 0.7546 (0.4423–1.2873) | 0.3014 |
| M | 1.5053 (0.8482–2.6716) | 0.1623 |
| RBC | 0.5884 (0.4021–0.8610) | 0.0063 |
| HB | 0.9810 (0.9689–0.9931) | 0.0023 |
| PLT | 1.0017 (0.9985–1.0050) | 0.2963 |
| GLU | 1.0933 (1.0132–1.1798) | 0.0215 |
| ALT | 1.0083 (0.9904–1.0266) | 0.3654 |
| AST | 1.0154 (0.9989–1.0320) | 0.0668 |
| DBIL | 1.0284 (0.9707–1.0895) | 0.3417 |
| IBIL | 0.9499 (0.9035–0.9986) | 0.0440 |
| ALB | 0.9062 (0.8566–0.9586) | 0.0006 |
| GLOB | 1.0493 (0.9962–1.1053) | 0.0694 |
| BUN | 1.1265 (1.0630–1.1938) | <0.0001 |
| Cr | 1.0038 (1.0013–1.0063) | 0.0027 |
| CysC | 1.4775 (1.1771–1.8546) | 0.0008 |
| Ka | 1.4772 (0.9582–2.2775) | 0.0773 |
| Na | 0.9475 (0.8802–1.0198) | 0.1506 |
| Ca | 0.3659 (0.0502–2.6662) | 0.3211 |
| PT | 1.0534 (0.9208–1.2050) | 0.4487 |
| INR | 1.3217 (0.3917–4.4595) | 0.6531 |
| PTA | 0.9879 (0.9729–1.0033) | 0.1222 |
| APTT | 1.0339 (0.9936–1.0759) | 0.1007 |
| TT | 1.0267 (0.8664–1.2166) | 0.7613 |
| FIB | 1.1513 (0.9693–1.3676) | 0.1085 |
| NLR | 1.0408 (0.9831–1.1019) | 0.1691 |
| MLR | 1.6554 (0.9450–2.8997) | 0.0780 |
| PLR | 1.0019 (0.9995–1.0042) | 0.1140 |
| SII | 1.0002 (1.0000–1.0005) | 0.1045 |
| SIRI | 1.0854 (1.0127–1.1634) | 0.0205 |
| AISI | 1.0004 (1.0001–1.0007) | 0.0136 |
| HALP | 0.9753 (0.9585–0.9924) | 0.0049 |
| PNI | 0.9117 (0.8664–0.9594) | 0.0004 |
| BAR | 1.1679 (1.0915–1.2496) | <0.0001 |
| NPAR | 1.1178 (1.0405–1.2008) | 0.0023 |
